# Inner Ear Dysfunction in Thyroid Disease: A Scoping Review

**DOI:** 10.3390/medicina61101793

**Published:** 2025-10-04

**Authors:** Athena Eliana Arsie, Luca Sacchetto, Carlotta Muneretto, Matteo Seno, Enrico Apa, Elisabetta Genovese, Daniele Monzani, Silvia Palma

**Affiliations:** 1 Otolaryngology-Head and Neck Surgery Department, University of Verona, 37129 Verona, Italy; athenaeliana.arsie@studenti.univr.it (A.E.A.); luca.sacchetto@univr.it (L.S.); carlotta.muneretto.cm@gmail.com (C.M.); matteo.seno@studenti.univr.it (M.S.); daniele.monzani@univr.it (D.M.); 2 Paediatric Audiovestibology Unit, Aziende Socio Sanitarie Territoriale dei Sette Laghi, 21100 Varese, Italy; it.apa.enrico@gmail.com; 3 Audiology, ENT Department, University of Modena and Reggio Emilia, 41100 Modena, Italy; elisabetta.genovese@unimore.it; 4 Audiology, Primary Care Department, Ausl Modena, 41121 Modena, Italy

**Keywords:** hearing loss, thyroid disease, autoimmunity, vertigo, vestibular disfunction

## Abstract

*Background and Objectives*: Sensorineural hearing loss (SNHL) is influenced by various causes, including thyroid diseases. For example, hypothyroidism and thyroid autoimmunity can damage the inner ear through hormonal, immune, and vascular mechanisms. Vestibular disorders like Ménière’s disease (MD) and benign paroxysmal positional vertigo (BPPV) also show possible associations with thyroid dysfunction. *Materials and Methods*: A review following PRISMA guidelines searched PubMed, Scopus, and Google Scholar for studies linking thyroid disorders with inner ear dysfunction. *Results*: Out of 985 screened records, 30 studies met inclusion criteria, involving various thyroid disorders, primarily hypothyroidism and autoimmune thyroiditis. Scientific evidence supports a correlation between hypothyroidism and hearing impairment. However, some studies also suggest a link between hyperthyroidism and inner ear disorders, particularly focusing on the role of autoimmunity in this context. Concerning vestibular dysfunction, the available studies are less abundant and support a significant association between thyroid disease and Meniere’s disease. *Conclusions*: There is a clear correlation between hypothyroidism and auditory function. A substantial body of literature also supports an association with vestibular disorders, although some discrepancies remain. Further research is needed to elucidate the underlying pathophysiological mechanisms (e.g., autoimmune, vascular, metabolic) involved with this correlation.

## 1. Introduction

Sensorineural hearing loss (SNHL) is a prevalent condition with a significant impact on quality of life, social interactions, and overall well-being [1,2,3]. The etiologies of SNHL are diverse and include genetic factors, noise exposure, aging (presbycusis), trauma, infections, use of ototoxic drugs, and various systemic diseases [4,5,6]. Among systemic conditions, thyroid diseases have long been hypothesized to play a role, although the nature and extent of this association remain a matter of debate, requiring further clarification [7,8]. Thyroid disorders, including both hypothyroidism and hyperthyroidism, are among the most common endocrine diseases worldwide [9,10]. It is well established that thyroid hormones are essential for the normal development of the auditory system and inner ear structures, with their absence or insufficiency during early life potentially leading to damage to the sensory components of the inner ear [11,12]. Historically, an association between thyroid disorders and hearing problems has been observed in both congenital (CH) and acquired hypothyroidism (AH) and occasionally in hyperthyroidism [8]. In addition to the well-known direct effect of thyroid hormones on the development and function of cochlear hair cells, several other hypotheses have been proposed. One such theory suggests that autoimmune mechanisms, mediated by antibodies such as anti-thyroid peroxidase (anti-TPO) and anti-thyroglobulin (anti-Tg), which are typically present in autoimmune thyroiditis, may impact the inner ear. For instance, immune-mediated inner ear disease (IMIED) is a recognized condition that can manifest as sensory neural hearing loss, which is often progressive and bilateral [1,6]. It has been reported that damage due to IMIED can occur directly as a result of an immune response toward inner ear antigens or indirectly by immune complex deposition in the inner ear organs, determining otologic and vestibular symptoms secondary to the endocochlear injury [13]. Vestibular function has been less frequently investigated; thus, a recent review on this topic concluded that vestibular dysfunction is likely underdiagnosed in patients suffering from IMIED [14].

Vascular insult has also been hypothesized as a possible contributor to inner ear damage. Thyroid dysfunction may compromise microcirculation, resulting in reduced cochlear blood flow [5]; further proposed mechanisms include metabolic disturbances and hypothermia, both associated with thyroid dysfunction. Moreover, the low body temperature commonly seen in hypothyroid patients may affect auditory brainstem responses (ABRs), potentially mimicking a retrocochlear pathology unrelated to the thyroid dysfunction itself [15]. As for vestibular disorders, such as Ménière’s disease (MD) and benign paroxysmal positional vertigo (BPPV), their association with thyroid diseases has also been explored. The relationship between BPPV and thyroid autoimmunity is likewise under investigation, with some studies suggesting a link between BPPV and Hashimoto’s thyroiditis (HT) independent of thyroid function status and others not confirming this association [16,17,18]. Given the variability of the findings, a review of the most recent literature is warranted to synthesize the available evidence. This scoping review aims to summarize current evidence, explore the proposed pathophysiological mechanisms, and highlight key methodological gaps in the existing literature to support future research and improve clinical management.

## 2. Materials and Methods

A literature search was conducted using the PubMed, Scopus, and Google Scholar databases to identify studies reporting on associations between thyroid disorders and inner ear dysfunction, including both audiological and vestibular conditions. This review was conducted in accordance with the Preferred Reporting Items for Systematic Reviews and Meta-Analyses (PRISMA) guidelines. The following search strings were used:

*(Thyroid Diseases [MeSH Terms] OR hypothyroidism OR hyperthyroidism OR Hashimoto disease OR Graves disease) AND (inner ear[MeSH Terms] OR inner ear disease OR cochlear disease OR vestibular disease OR hearing loss OR vertigo OR Meniere disease) AND (“2005/01/01”[Date-Publication]: “2025/12/31”[Date-Publication])*.
*((hypothyroidism OR autoimmune thyroiditis OR Hashimoto disease) AND (inner ear disease OR cochlear disease OR vestibular disease OR hearing loss OR vertigo OR presbycusis OR Meniere disease OR “recurrent vertigo” OR “benign paroxysmal positional vertigo”)) AND (“2005/01/01”[Date-Publication]: “2025/12/31”[Date-Publication])*.

Subsequently, the full text of selected studies was screened for final selection. All studies identified by the initial literature search were reviewed independently by three authors (AEA, CM, and MS). All titles and abstracts were assessed. Studies published within the time frame of 2005 to 2025 were included, to ensure the selection of literature based on contemporary diagnostic criteria and clinical practices, thereby enhancing the relevance and applicability of the findings to current clinical contexts.

Studies were eligible for inclusion if they met all of the following criteria: (I) original observational studies (cohort, case–control, or cross-sectional) or experimental studies (randomized controlled trials); (II) case series including at least 5 patients; (III) diagnosis of thyroid disease confirmed by laboratory testing; and (IV) diagnosis of audiological and/or vestibular disorders confirmed by instrumental examination. Exclusion criteria were as follows: (I) articles not published in English; (II) single case reports, letters to the editor, or commentaries; (III) studies without full-text availability; (IV) lack of laboratory confirmation of thyroid disease or absence of audiological and vestibular testing to confirm inner ear disorders; (VI) genetic syndromes; and (VI) animal or in vitro studies.

**Figure 1:**
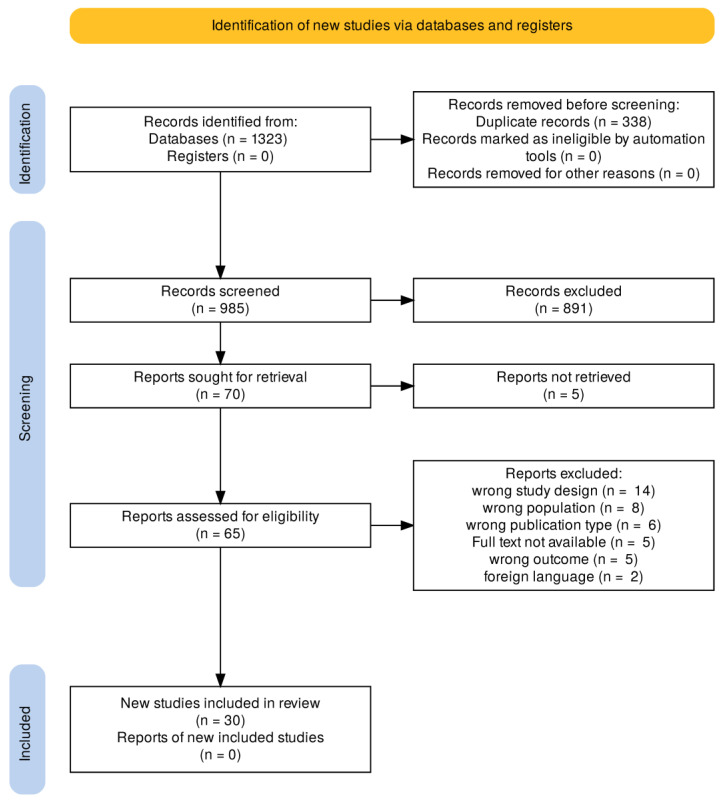
Selection process.

The selection process is summarized in Figure 1.

## 3. Results

A total of 1323 records were initially identified through database searching. After removing duplicates, the remaining 985 records were assessed. Of these, 70 full-text articles were selected for further evaluation. Full texts were then assessed according to predefined inclusion and exclusion criteria. Discrepancies in the selection process were resolved through discussion or, when necessary, by consulting a fourth senior reviewer (DM). A total of 30 articles were selected for inclusion. For each study, we extracted key information such as authorship, country of origin, year of publication, sample size, and main findings. After the screening process, abstracts and full texts were evaluated. A summary of these articles is presented in Table 1.

Sixteen were case–control studies, three were prospective, and eleven had a retrospective design. All studies involved adult patients, and a few of them [9,11,29,30] also investigated pediatric populations.

Of the thirty studies included, ten (33%) focused on autoimmune hypothyroidism (Hashimoto’s thyroiditis or autoimmune thyroiditis), while another ten (33%) addressed hypothyroidism of various etiologies (iatrogenic, congenital, or non-autoimmune acquired). Eight studies examined multiple thyroid disorders without distinction. Only two studies investigated patients with hyperthyroidism. Six studies addressed peripheral vestibular dysfunction, four focused on sudden sensorineural hearing loss (SSNHL), four on MD, and one on tinnitus. Twenty studies enrolled fewer than 100 patients, with variables related to patient comorbidities in five of the studies. Most articles reported short follow-up periods. Some studies reported a higher prevalence of MD among patients with hypothyroidism, suggesting a possible therapeutic benefit from thyroxine supplementation [17,18]. Due to the methodological heterogeneity evidenced, we classified the articles by their main focus, including six types: hypothyroidism and auditory function, hyperthyroidism and auditory function, autoimmune thyroid diseases and auditory–vestibular dysfunction, sudden sensorineural hearing loss, vestibular disorders, and tinnitus.

## 4. Discussion

### 4.1. Hypothyroidism and Auditory Function

Hearing impairment has been reported in hypothyroidism [34], endemic cretinism [35], Pendred syndrome, and in patients with a resistance to thyroid hormones. Thyroid hormones exert multiple physiological effects [36], such as protein synthesis; cell growth and differentiation; thermogenesis; and the maturation of the central nervous, musculoskeletal, and epidermal systems. While it is well established that CH causes physiological, morphological, and developmental abnormalities in the auditory system, the impact of AH and the effectiveness of hormone replacement therapy (HRT) on hearing function remain controversial. Tatlıpınar et al. [7] observed a negative correlation between baseline FT4 levels and hearing impairment, suggesting that disease severity may influence the extent of the auditory dysfunction. Moreover, patients with lower FT4 levels and higher TSH levels showed a poorer improvement in pure tone audiometry (PTA) after HRT, indicating that hormone replacement may not yield significant auditory improvements in cases of severe hypothyroidism. However, this study population was limited in size. These findings align with those of Hashemipour et al. [9], who, in the context of a stringent screening program for CH and the prompt initiation of therapy, found a very low prevalence of hearing loss in affected patients. A distinction must be made between acute and chronic hypothyroidism, associated with a gradual onset and long-standing thyroid dysfunction. Concerning acute disease, Psaltakos et al. [29] reported a significant elevation of hearing thresholds in patients who had undergone a total thyroidectomy and had not yet received a levothyroxine replacement, compared to controls.

Distortion Product Otoacoustic Emission (DPOAE) responses, although still present in CH patients, showed reduced amplitudes, particularly in the mid-frequency range, when compared to healthy individuals [11]. This suggests that the bio-electromechanical functioning of the cochlea may be compromised in this population. Similarly, it has been [8] reported an increase in the wave V latency in the auditory brainstem evokes potentials (ABR). Hypothyroid patients more frequently exhibited an absent or reduced amplitude of the OAE compared to the control group. Notably, these audiological abnormalities were not correlated with serum TSH or FT4 levels, which is consistent with findings by Grimmichova et al. [3], who found no hearing impairment in adults with acquired hypothyroidism. The results point toward a likely subclinical cochlear dysfunction and a predisposition to metabolic presbycusis, especially in patients with frequent fluctuations in serum thyroid hormone levels during follow-up.

The analysis of the literature allows us to conclude that CH clearly affects hearing function, manifesting as cochlear symptoms such as tinnitus and hearing loss [30]. Conversely, the impact of AH remains a matter of debate, as it may vary based on whether the condition is acute or chronic, the severity of the hormonal deficiency, and FT4 levels. In the future, it will be important to investigate the long-term effects of hypothyroidism corrections through replacement therapy, as well as to explore how hormonal fluctuations influence cochlear function, with a particular focus on the implementation of large sample sizes. Prospective studies with long follow-ups would allow us to clarify the role of replacement therapy.

### 4.2. Hyperthyroidism and Auditory Function

As mentioned, thyroid hormones are essential for the development and proper functioning of the auditory system, including cochlear and vestibulocochlear nerve myelination [19]. A few studies have focused specifically on the hyperthyroid pathology. While Trivedi reported that hearing loss can occur in both hypothyroidism and hyperthyroidism [10], in another study it was explicitly stated that subjects presenting with hyperthyroidism did not show significant differences in pure tone audiometry or ABR results compared to healthy controls. However, this was a retrospective study with only 14 patients [15]. On the other hand, [19] patients with Graves’ disease (GD) demonstrated significantly higher hearing thresholds across all frequencies compared to healthy controls, with a notable hearing loss at high frequencies (4000 and 8000 Hz). The authors suggested that the metabolic effects of thyroid hormones and thyrotoxicosis might play a role and that autoimmunity, although not directly correlated with Thyroid-Stimulating Hormone Receptor Antibody (TRAb) levels, could be an influencing factor [19]. Mutlu et al. [31] proposed an indirect mechanism: they found that high doses of levothyroxine (L-T4), often administered post-thyroidectomy in thyroid cancer patients and capable of inducing a subclinical hyperthyroid state or affecting bone mineral density, were associated with hearing loss. The likely underlying cause was systemic skeletal demineralization, which includes the temporal bone and cochlea. Sensorineural hearing loss was predominantly high-frequency, though conductive loss was also reported [31]. Overall, we observed a significant imbalance between the number of studies on hypothyroidism and the relative scarcity of those addressing hyperthyroidism. This finding indirectly suggests the predominant role of hypothyroidism on hearing disfunction but indicates that it is worthwhile to further investigate the correlation between vestibulocochlear dysfunction and hyperthyroidism, particularly as it relates to autoimmunity.

### 4.3. Autoimmune Thyroid Diseases and Auditory–Vestibular Dysfunction

Although the pathophysiology of SNHL remains poorly clarified, the hypothesis of an immune-mediated mechanism has become one of the most widely accepted theories over the past three decades [37]. Several autoimmune diseases, such as systemic lupus erythematosus [38], systemic sclerosis [39], rheumatoid arthritis [40], and ankylosing spondylitis [41], have been associated with hearing loss. Disseminated vasculitis [42] and celiac disease [43] have also been linked to impaired auditory function. Rodríguez-Valiente et al. conducted a study on patients with SNHL not attributable to presbycusis and identified 15 individuals with AITD, mainly HT [6]. In these patients, hearing loss was often bilateral and most frequently presented as sudden SNHL. Despite ongoing levothyroxine replacement therapy, corticosteroid treatment led to a hearing improvement in over half of the cases. This suggests the presence of IMIED associated with AITD, thus supporting the need for a targeted immunosuppressive therapeutic approach in these patients [6]. Arduc et al. [2] conducted a case–control study in which patients with HT and elevated autoantibody levels exhibited significantly higher hearing thresholds, despite having TSH, fT3, and fT4 levels within the normal range. Auditory thresholds in euthyroid patients with autoimmune thyroid disease (AT) were impaired across the entire auditory spectrum, with an early and more prominent involvement of high frequencies [25]. A subset of these patients also experienced episodes of endolymphatic hydrops of indeterminate origins. Given the euthyroid state, both findings suggest a relationship with the presence of anti-thyroid antibodies [25]. Gunes et al. [1] evaluated the impact of thyroid antibodies on audiometric outcomes by comparing affected individuals with healthy controls and found that thyroid antibody positivity may lead to inner ear dysfunction. As the degree of auditory involvement tends to increase with the duration of autoantibody exposure, the close monitoring of auditory response variations over time is warranted. Taken together, the evidence suggests that hearing loss may be more directly related to the autoimmune disorder itself and the presence of circulating antibodies rather than to its management with levothyroxine. There is the lack of studies specifically focusing on the metabolic effects of autoimmunity on the terminal circulation of the inner ear, and how these mechanisms are triggered or modulated by an underlying autoimmune substrate remains unclear.

### 4.4. Sudden Sensorineural Hearing Loss and Thyroid Disease

Sudden sensorineural hearing loss (SSNHL) is a common and alarming condition that often prompts urgent audiologic evaluation. The etiology of SSNHL remains unclear, though it is generally believed to involve viral infections, vascular events, autoimmune phenomena, and other contributing factors. From an immunological perspective, the study by Sun et al. suggests that thyroid autoimmunity may be a pathogenic factor in SSNHL, as they found that 24.8% of SSNHL patients had elevated thyroid autoantibodies [32]. Their investigation focused specifically on TPOAb and TgAb, hypothesizing that these autoantibodies might exert direct immune-mediated damage to the terminal vascular structures of the inner ear, potentially through oxidative stress mechanisms. Similar results have been found by the large-scale nationwide study by Tsai, who reported that a pre-existing thyroid disease, both hypothyroidism and hyperthyroidism, was associated with an increased risk of SSNHL [12]; similare results were also found by Oiticica [20] and Kim [23].

The shared finding across these studies is the growing evidence that thyroid disorders, both autoimmune and functional (hypothyroidism, hyperthyroidism, goiter), represent significant risk factors for sudden sensorineural hearing loss. This suggests carefully evaluating the clinical history of these subjects, also taking in consideration thyroid functionality. These associations may reflect an immune, vascular, or metabolic pathogenesis affecting the inner ear.

### 4.5. Vestibular Disorders and Thyroid Diseases

Several studies have been conducted to investigate the potential role of thyroid diseases in vestibular disorders. Chiarella et al. [26] documented a significant correlation between subclinical vestibular impairment and anti-TPO antibody titers in euthyroid patients with HT, highlighting that circulating antithyroid autoantibodies may represent a risk factor for developing vestibular dysfunction [26].

An autoimmune contribution has also been hypothesized in the pathogenesis of Ménière’s disease (MD). A significantly higher prevalence of thyroid autoimmunity was observed compared to two control groups (healthy volunteers and patients with acute unilateral peripheral vestibulopathy) [21]. The main autoantibodies detected were TPO-Ab, Tg-Ab, and TR-Ab. The diffusion of immune complexes into the inner ear or the development of autoimmune microangiopathy within the labyrinth potentially alters the endolymph composition and stimulates vestibular sensory cells, thereby triggering vertigo attacks. These findings support the hypothesis that thyroid autoimmunity may contribute to the pathogenesis of MD, similarly to the results from a large Korean study [22]. Consistently, Lin et al. found that the prevalence of MD was significantly higher in patients with hypothyroidism, especially in women over the age of 50 [28].

Santosh reported an improvement in the clinical symptoms of MD following the treatment of hypothyroidism [27]. This study underscores the importance of endocrine screening in MD patients, to identify those at higher risk, especially since vestibular symptoms may improve following hormonal correction. One of main questions concerning MD is that the diagnosis is produced clinically, and sometimes it is difficult to distinguish Menière’s disease from other types of vertigo that might occur independently with hearing loss and tinnitus. We can assume that a consensus regarding the association between peripheral vertigo and thyroid dysfunction exists except for BPPV. In this case the literature appears less consistent. Papi et al. reported a strong association between BPPV and hypothyroidism (mainly subclinical) as well as autoimmune chronic thyroiditis [16]. On the other hand, two studies by Miśkiewicz-Orczyk did not confirm any influence of thyroid metabolism on cVEMP results or on the directional preponderance in the caloric test [5,33]. Similarly, Sari et al. found no statistically significant differences in thyroid autoantibody levels among patients with BPPV, those with non-BPPV, and healthy controls, concluding that no relationship could be established in their study [17]. These discrepancies may also be attributable to differences in the study populations (e.g., Chiarella included asymptomatic patients, whereas Miśkiewicz-Orczyk focused on those with already diagnosed chronic vertigo) and the specific vestibular conditions examined. Moreover, both studies included a small sample size; acute cases were not evaluated, and the peripheral vertigo cases analyzed were not homogeneous. Tricarico et al. [44] showed that HT was associated with a recurrence of BPPV, and a significant correlation was found with level of serum TPO-Ab and TG-Ab but not with thyroid hormones. This latter report was a large sample study, but no controls were examined, and a long-term follow-up of cases was not adopted, so the data about the recurrence of BPPV and thyroid autoimmunity are not sufficient to draw any definite conclusions.

In conclusion, this review of the literature evidenced a substantial agreement on the correlation between hypothyroidism and autoimmune thyroid disease and vestibular disorders, except for BPPV. As BPPV is considered a mechanical disorder caused by otoconia dislodged from the utricle into the semicircular canals, probably, due to the peculiarity of the disease, a correlation, when one exists, is difficult to demonstrate.

### 4.6. Tinnitus and Thyroid Disorders

Regarding the association between thyroid disease and tinnitus, the literature is scarce. Only one study met our inclusion criteria [24] and demonstrated a relationship between hypothyroidism and an increased risk of tinnitus. The study was based on data from the Taiwan National Health Insurance Research Database, and the authors found that hypothyroid patients were at higher risk of developing tinnitus in the presence of comorbid conditions such as vertigo, hearing loss, and insomnia. They proposed that thyroid hormones may affect cochlear maturation, interact with adrenergic receptors, and influence peripheral blood flow and the sympathoadrenal system regulating cochlear perfusion. The disruption of this balance could result in hypoxic insult, manifesting clinically as tinnitus. As tinnitus is the sensation of hearing a sound in the absence of an external source and in most cases is associated with sensorineural hearing loss, the pathogenesis in cases of thyroid disease appears to be strongly correlated with one of the hearing impairment.

This review has a few limitations. Gray literature, conference abstracts, and unpublished data were not addressed. Moreover, many studies included small sample sizes (twenty studies enrolled fewer than 100 patients) and short follow-up periods, particularly for audiological outcomes in cases of autoimmune thyroiditis and in studies evaluating hearing function after the hormonal correction of hypothyroidism. Another limitation is tied to the presence of confounding variables related to patient comorbidities in five of the studies.

## 5. Conclusions

In conclusion, there is a clear correlation between hypothyroidism, thyroid autoimmune disease, and auditory function. Links with hyperthyroidism are much weaker. Studies including larger sample sizes and longer follow-up periods are necessary. A substantial body of literature also supports an association with vestibular disorders, although some discrepancies remain.

Some authors have indicated, the appropriate treatment of thyroid dysfunction provides clinical benefits for the symptoms related to inner ear diseases as well; this finding should be confirmed by large-scale studies. There is a lack of studies focused on a deep analysis of the pathogenesis underlying audio–vestibular alterations associated with thyroid dysfunctions, including biochemistry and molecular analyses as well.

## Figures and Tables

**Table 1:** Summary of the articles selected.

Author and Year	Country	Type of Study	Sample Size	Inner Ear Pathology	Thyroid Pathology	Main Findings
**Gunes, 2017 [1]**	Turkey	Case–control	17 patients with short-term antibody positivity 15 patients with long-term antibody positivity 18 controls	Hearing impairment	Autoimmune thyroiditis	Subjects with long-term thyroid antibody positivity had significantly worse hearing thresholds (500–4000 Hz).
**Arduc A, 2015 [2]**	Turkey	Case–control	30 patients with Hashimoto’s thyroiditis 30 controls	Hearing impairment	Hashimoto’s thyroiditis (HT)	HT patients had significantly worse hearing at 250, 500, and 6000 Hz. Thresholds at 250 and 500 Hz positively correlated with anti-thyroglobulin antibody levels.
**Grimmichova, 2025 [3]**	Czech Republic	Case–control	30 patients with hypothyroidism 30 controls	Hearing impairment	Acquired hypothyroidism	No significant differences in hearing test results. Mild hearing impairment, common, was not linked to acquired hypothyroidism in this adult population.
**Miśkiewicz-Orczyk K, 2022 [5]**	Poland	Retrospective	28	Peripheral vertigo	Hashimoto’s thyroiditis	In HT women with chronic vertigo, normal hearing was present in 54%, BPPV was the most common vertigo cause. Vestibular hyporeflexia was found in 54%. No correlations with thyroid function were observed.
**Rodríguez-Valiente A, 2018 [6]**	Spain	Retrospective	220	Hearing Impairment	Autoimmune thyroid disease (AITD)	Sudden bilateral SNHL is common in AITD patients despite adequate thyroid hormone therapy. Corticosteroid treatment improved hearing in several cases, indicating a possible immune-mediated mechanism.
**Tatlıpınar A, 2023 [7]**	Turkey	Prospective	50	Hearing impairment	Acquired hypothyroidism (AH)	Lower baseline free T4 levels correlated with worse hearing. Hypothyroidism severity predicted reduced hearing improvement after levothyroxine treatment.
**Pinheiro dos Santos KP, 2010 [8]**	Brazil	Case–control	30 patients with hypothyroidism 30 controls	Hearing impairment	Acquired hypothyroidism (AH)	Women with AH showed auditory abnormalities, suggesting subclinical cochleovestibular dysfunction independent of thyroid hormone levels.
**Berker, 2011 [19]**	Turkey	Case–control	22 patients with Graves’ disease 22 controls	Hearing impairment	Graves’ disease (GD)	GD patients exhibited higher hearing thresholds at 4000 and 8000 Hz. Hearing loss at 8000 Hz was strongly linked to elevated fT3/fT4 and suppressed TSH, suggesting thyrotoxicosis-related auditory impairment.
**Oiticica J, 2010 [20]**	Brazil	Retrospective	166	Sudden sensory neural hearing loss (SSNHL)	Multiple metabolic disorders	Thyroid hormone abnormalities were present in 21.6% of SSNHL patients. Results suggest hyperglycemia and thyroid dysfunction are common and may be key risk factors for sudden hearing loss.
**Fattori B, 2007 [21]**	Italy	Case–control	50 patients with Ménière’s disease (47 unilateral, 3 bilateral)	Ménière’s disease	Thyroid autoimmunity	A significant association was found between MD and thyroid autoimmunity. TPO antibodies were most common, and 14% of MD patients were hyperthyroid.
**Hashemipour, 2010 [9]**	Iran	Case–control	94 patients with congenital hypothyroidism (CH) 450 controls	Hearing impairment	Congenital hypothyroidism (CH)	SNHL was detected in 3.2% of CH cases versus 0.2% controls. Hearing loss seems to be linked to thyroid dyshormonogenesis, suggesting genetic involvement.
**Trivedi, 2023 [10]**	India	Retrospective	50	Hearing impairment	Multiple thyroid disorders	Hearing loss affected 37.5% of hypothyroid and 30% of hyperthyroid patients, predominantly sensorineural at high frequencies, with mild conductive loss also in hypothyroidism.
**Kim, 2020 [22]**	China	Case–control	8183 patients with Ménière’s disease 32,732 controls	Ménière’s disease	Multiple thyroid disorders	Ménière’s disease patients showed higher rates of goiter, hypothyroidism, and hyperthyroidism, with associations confirmed after adjusting for multiple factors.
**Oliveira de Andrade CL, 2019 [11]**	Brazil	Case–control	50 patients with congenital hypothyroidism (CH) 42 controls	Cochlear disfunction	Congenital hypothyroidism (CH)	Children with CH showed reduced Distortion Product Otoacoustic Emission (DPOAE) amplitudes, especially mid frequencies; cochlear function correlated weakly with disease duration, diagnosis age, hormone irregularities, and treatment status.
**Tsai YT, 2020 [12]**	Taiwan	Case–control	3331 patients with sudden SNHL 13,324 controls	SSNHL	Pre-existing thyroid disfunction	In 3331 SSNHL patients, thyroid diseases were more frequent than controls; hypothyroidism increased SSNHL risk in those over 50, hyperthyroidism in women.
**Kim SY, 2020 [23]**	Korea	Retrospective	8658 patients with sudden SNHL 34,632 controls	SSNHL	Pre-existing thyroid disease	Goiter and hypothyroidism were significantly more common in SSNHL patients, suggesting these as contributing risk factors.
**Hsu, 2022 [24]**	China	Retrospective	6062	Tinnitus	Hypothyroidism	Hypothyroid patients showed a significantly higher tinnitus risk than controls, consistent across age, sex, and comorbidities, with age also identified as an independent risk factor.
**Álvarez Montero OL, 2021 [25]**	Spain	Case–control	128 treated Hashimoto’s patients 128 untreated Hashimoto’s patients 209 healthy controls	Hearing impairment	Autoimmune thyroiditis	Women with HT, treated or untreated, showed significant hearing loss, especially at 8–20 kHz, suggesting auditory damage. Younger patients showed high-frequency loss, while older patients had broader impairment.
**Chiarella, 2014 [26]**	Italy	Case–control	47 patients with Hashimoto’s thyroiditis (HT) 21 patients with multinodular goiter (MNG) 30 controls	Vestibular disorders	Thyroid autoimmunity	In euthyroid HT patients, over half showed vestibular test abnormalities despite no vertigo, correlating with elevated TPO antibodies, suggesting thyroid autoimmunity may cause subclinical vestibular dysfunction.
**Thornton, 2007 [15]**	UK	Retrospective	14 patients with hyperthyroidism 21 patients with hypothyroidism	Hearing impairment	Hyperthyroidism and hypothyroidism	In hypothyroid patients, hearing loss and ABR abnormalities resolved with thyroxine treatment and normalized body temperature, indicating low temperature, not retrocochlear pathology, as the likely cause.
**Santosh, 2016 [27]**	India	Retrospective	35	Ménière’s disease	Hypothyroidism	In 35 Ménière’s patients, 12 had hypothyroidism; all improved with thyroxine treatment, suggesting a significant link between hypothyroidism and MD’s symptoms.
**Lin, 2019 [28]**	China	Retrospective	5410	Ménière’s disease	Hypothyroidism	MD was more prevalent in hypothyroid patients, especially over 50, but thyroxine treatment reduced the risk to that of non-hypothyroid individuals.
**Psaltakos, 2012 [29]**	Greece	Prospective	52	Cochlear disfunction	Acute hypothyroidism	In thyroid cancer patients post-thyroidectomy without hormone replacement, acute hypothyroidism led to elevated hearing thresholds and reduced cochlear function, with significantly worse auditory performance than healthy controls.
**Singh, 2018 [30]**	India	Case–control	50 patients with hypothyroidism 50 controls	Hearing impairment	Acquired hypothyroidism	Hypothyroid patients had frequent cochleo-vestibular symptoms, elevated thresholds, and ABR abnormalities. After thyroxin treatment, 46.42% showed hearing improvement and increased wave Va amplitude, indicating functional recovery.
**Mutlu, 2024 [31]**	Turkey	Prospective	62	Hearing impairment	High-dose levothyroxine (L-T4)	High-dose L-T4 therapy in post-thyroidectomy patients was linked to increased bilateral and sensorineural hearing loss, osteopenia, and osteoporosis compared to lower doses, independent of iodine treatment.
**Sun XM, 2022 [32]**	China	Retrospective	105	Sudden SNHL	Autoimmune thyroiditis	Thyroid antibody-positive patients showed more severe high-frequency hearing loss, but both antibody-positive and -negative groups improved after treatment, suggesting autoimmunity’s role in SSNHL.
**Papi, 2009 [16]**	Italy	Case–control	134 patients with BPPV 100 controls	BPPV	Autoimmune chronic thyroiditis	BPPV patients had higher TSH, thyroid antibodies, and subclinical hypothyroidism (21%), with 34% showing autoimmune thyroiditis versus 2% in controls, indicating a strong association.
**Miśkiewicz-Orczyk K, 2022 [33]**	Poland	Retrospective	28	Peripheral vertigo	Hashimoto’s thyroiditis	In female patients with BPPV, MD, or vestibular neuronitis, vestibular test results showed no significant correlation with thyroid hormones, antibodies.
**Sari K, 2015 [17]**	Turkey	Case–control	50 patients with BPPV 50 patients with non-BPPV 60 healthy controls	BPPV	Hyperthyroidism	Normal TSH levels and findings suggest no significant association between BPPV and thyroid autoimmunity.
**Choi, 2021 [18]**	Korea	Case–control	19,071 patients with BPPV 76,258 controls	BPPV	Multiple thyroid disorders	Thyroid disorders were more common in BPPV patients, but levothyroxine treatment was not associated with increased BPPV risk.

## Data Availability

No new data were created or analyzed in this study.
